# Priming Behavioral Control Enhances Sharing in Preschoolers

**DOI:** 10.3389/fpsyg.2022.892382

**Published:** 2022-07-08

**Authors:** Chanmi Lee, Hyun-joo Song

**Affiliations:** ^1^Assesta Co., Ltd., Seoul, South Korea; ^2^Department of Psychology, Yonsei University, Seoul, South Korea

**Keywords:** sharing, fairness, prosocial behavior, behavioral control, priming

## Abstract

Although young children demonstrate knowledge of fairness norms, their actual sharing is often inconsistent with their understanding. A possible explanation for this discrepancy is the failure of behavioral control in young children. Thus, the present research manipulated behavioral control experimentally and examined its effect on the sharing behavior in 3- to 4-year-olds (*N* = 64). Children were randomly assigned to either the behavioral control or the neutral prime conditions. In the behavioral control prime condition, the children listened to a story in which a protagonist exerted behavioral control actively, refraining from eating candies. In the neutral prime condition, the children listened to a story in which a protagonist did not explicitly engage in behavioral control. The children then participated in the dictator game. The experimenter asked the children to share as many stickers as they wanted or should with an anonymous child. Children in the behavioral control prime condition shared more stickers than those in the neutral prime condition. However, the two groups did not differ in their judgments of fairness and emotional experiences. The current research provides evidence that preschoolers’ sharing behaviors can be facilitated by behavioral control.

## Introduction

Children exhibit sensitivity to fairness early in their lives. Thus, children who are 3 years old or older can explicitly state that they endorse the norm of fairness when sharing resources with others (Olson and Spelke, 2008; Smith et al., 2013). Moreover, infants demonstrate sensitivity to fair distribution in implicit behavioral measures. During the second year of life, they expect an agent to allocate resources equally between two individuals (Schmidt and Sommerville, 2011; Sloane et al., 2012) and prefer fair to unfair distributors (Geraci and Surian, 2011; Lucca et al., 2018). Recently, investigators have begun to reveal that even infants as young as 1 year of age expect equal resource allocation (Buyukozer Dawkins et al., 2019).

Although young children understand fairness very early, they often do not adhere to fairness norms when given a chance to share with others. For example, when asking 3- to 4-year-olds to divide resources between themselves and a recipient, they often favor themselves, giving less than half of the resources to another (Benenson et al., 2007; Fehr et al., 2008). Only by about age 7–8 years do children share equally between themselves and a recipient (Fehr et al., 2008; Smith et al., 2013).

How can we account for the gap between young children’s fairness knowledge and sharing behavior? One potential explanation for such a gap is young children’s failure in behavioral control when producing sharing behavior (Blake, 2018). Behavioral control refers to the ability to modulate behavior in pursuit of attaining one’s goals (Kopp, 1982; Karoly, 1993). Social norms often involve conflict between benefiting others and maximizing immediate self-interest (Buckholtz, 2015). Behavioral control is required to ride out selfish desires and conform to social norms. For example, after participating in an inhibition task, adult participants with depleted behavioral control are more likely to violate social norms than those without it (DeBono et al., 2011). Similarly, when children share resources, aligning their behavior with fairness norms may require behavioral control. That is, children may need to curb temptation to have all resources for themselves (Blake et al., 2015). Thus, young children may fail to share equally because of their difficulty in behavioral control.

Previous studies suggest that behavioral control plays a critical role in closing the children’s knowledge–behavior gap. In 6- to 13-year-olds, there is a correlation between behavioral control reported by parents and the gap between stated norms and actual sharing in the dictator game (Blake et al., 2015). Such a link between behavioral control and sharing has also been suggested for preschoolers. For example, researchers used an experimental task (i.e., day–night task) to measure behavioral control in children aged 4–6 years. They found that 4–6-year-olds’ behavioral control was associated with sharing at least one item (Aguilar-Pardo et al., 2013) and self-disadvantageous sharing (Xie et al., 2019). In addition, parents reported that behavioral control at 2 years was related to children’s sharing at 5 years (Paulus et al., 2015). However, there is conflicting evidence regarding this. Smith et al. (2013) found that 3–8-year-olds’ tendency to share equally in the dictator game was not associated with behavioral control measured in bear–dragon and day–night tasks (Smith et al., 2013). Such discrepancies in the literature could be due to the use of correlational research that employs different measures. Correlational studies are the basis for most of the evidence from children, probably because an experimental paradigm to manipulate behavioral control in children has not been established until recently. These correlational findings provide insufficient evidence on the mechanism through which behavioral control affects children’s sharing actions.

Some studies have experimentally manipulated behavioral control in a laboratory and revealed evidence for the relationship between behavioral control and children’s sharing behaviors. Depleting behavioral control capacities through a stop-signal reaction time task that required the inhibition of a prepotent response decreased 6–9-year-olds’ sharing in a subsequent dictator game (Steinbeis, 2018). In addition, the same population was more likely to share resources following exposure to stories that primed behavioral control (e.g., a protagonist resisting the temptation to eat treats) than after listening to neutral stories unrelated to behavioral control (Steinbeis and Over, 2017). However, there is limited evidence on the causal relationship between behavioral control and sharing among younger children. To the best of our knowledge, the only piece of experimental evidence from children younger than 6 years is that of Liang et al. (2020). In that study, researchers found that 5–6-year-olds exposed to the behavioral control prime shared more resources than those exposed to the neutral prime.

However, the causal role of behavioral control in sharing has not been explored in children under 5 years of age. Children younger than 5 rarely share their own resources with others equally (Lane and Coon, 1972; Benenson et al., 2007; Fehr et al., 2008), thus showing a robust gap between fairness norms and sharing (Smith et al., 2013). Revealing whether priming behavior control induces less selfish sharing behaviors in children under five is essential to the investigation of the nature of early prosocial tendencies. If not only older children’s but also younger children’s sharing requires behavioral inhibition, it could suggest that children’s sharing decisions are not automatic and effortless processes, in contrast to the view that humans are intuitively cooperative (Zaki and Mitchell, 2013; Rand, 2016) even early in development (Warneken and Tomasello, 2006; See Chajes et al., 2022 for a similar argument).

The present research aimed to examine the causal relationship between behavioral control and sharing in 3–4-year-old children. This age group was chosen because previous research examined the role of behavioral control only in 5-year-old and older children’s sharing behaviors, and 3- to 4-year-olds were the youngest age group tested in the dictator game (Blake and Rand, 2010). We used a priming paradigm to experimentally manipulate behavioral control. In priming paradigms, participants are typically exposed to stimuli that activate mental representations and guide subsequent responses (Bargh and Chartrand, 2000; Bargh, 2006). In the current research, adapting the priming paradigm used in Steinbeis and Over (2017), children listened to a story about a protagonist who did or did not actively exert behavioral control. In the dictator game following the story, the experimenters asked half of the children to share as many items as they wanted, and the other half to share as many items as they should with an anonymous child.

The dictator game has been widely used to measure children’s fairness considerations when sharing resources with others (Gummerum et al., 2010; Smith et al., 2013). It is a very simple and tightly controlled experimental procedure considered a valid method in measuring children’s fair or prosocial sharing in various cultures (Ibbotson, 2014; Rajhans et al., 2016). In a typical dictator game, a participant is given some valued resources and asked to divide them between himself/herself and another person (or receiver), and they are anonymous to each other. Previous research using the dictator game paradigm with young children has revealed that with increasing age, children become fairer distributors (e.g., Benenson et al., 2007; McAuliffe et al., 2017).

When asking children to share as they should, the request could invoke norms that should be followed and thus, may have different influences on sharing behavior than asking them to share as they want. Children aged 6 to 9 years shared more when told to share as they should than when directed to share as they wanted (Steinbeis and Over, 2017). If sharing instruction affects preschoolers’ sharing, the effect of priming behavioral control may be different between the two sharing contexts. The experimenters also asked the children to indicate how they felt and rate whether they thought each distribution of the seven monetary units was fair. In this regard, researchers examined whether prosocial behaviors could be affected by priming stories on children’s emotional experiences and fairness judgments (Steinbeis and Over, 2017).

The present study also examined whether the effect of priming behavioral control on children’s sharing decisions could be mediated by their emotional states or fairness judgment. However, we made several procedural modifications from Steinbeis and Over (2017) to measure changes after priming behavioral control in younger children’s emotional experiences and fairness judgment behavioral controls. First, we asked the children to rate their emotions immediately after priming to detect subtle emotional state changes. However, Steinbeis and Over (2017) asked the children about their emotional experiences after completing the dictator game. Because of the delay between priming and emotion rating, the measure might not reflect exactly how the children felt immediately after priming. Second, we modified the emotion and fairness ratings to make it easier for younger children. Steinbeis and Over (2017) asked children how weakly or strongly they experienced each specific emotion (happy, sad, and angry). Instead, we asked our children to indicate the valence of the experienced emotions (positive, neutral, or negative emotions). Regarding fairness ratings, Steinbeis and Over (2017) presented children with several ways of distribution (7:0, 6:1, 5:2, and 4:3) and asked them to judge whether each distribution was fair. However, the researchers did not provide information about the distributor and the recipient, which might have confused the children: Children might have thought of themselves as distributors or recipients, not as third-party judges when judging each distribution. In the present study, we explicitly informed the children that the other child could share seven stickers with an anonymous child, and asked how many stickers they thought were good for the other child to share.

We hypothesized that priming behavioral control would lead to more generous sharing among young children. Thus, it was predicted that participants would share more stickers after listening to the behavioral control story compared to the neutral story and that participants’ emotion and fairness ratings would not differ between the behavioral control prime and neutral prime conditions.

## Materials and Methods

### Participants

Participants were 64 children aged 3 to 4 years old ( $M_{age}$ = 48.23 months, *SD* = 4.14, range = 42.20–57.40; 31 girls). *A priori* power analysis indicated that a minimum of 52 subjects were required to have 80% power for a large sized effect when employing the traditional 0.05 criterion of statistical significance. We tested an additional 15 children but excluded them from the final sample. The reasons for exclusion included failure to count the number of stickers used in a dictator game (*n* = 9), failure to understand the task (*n* = 1), participation in the task (*n* = 2), or inattention during the procedure (*n* = 3). We recruited participants in Seoul, South Korea, and the surrounding areas through advertisements in online parenting communities. This study was approved by the institutional ethics review board of Yonsei University. Before testing the participants, written consent was obtained from their parents. No language or cognitive delay was reported by the parents. No specific demographic data were collected; however, all the participants were from monolingual Korean-speaking families who resided in Seoul metropolitan areas and had mostly middle-class backgrounds.

### Design

The present study used a 2 (prime condition: behavioral control prime condition vs. neutral prime condition) × 2 (sharing instruction: want vs. should) between-subject design. Children heard a story during the priming process and then participated in the dictator game task. Children were randomly assigned to either the behavioral control or the neutral prime condition. In the behavioral control prime condition, the children listened to a story in which the protagonist exerted behavioral control (*n* = 32; 16 girls). In the neutral prime condition, the children listened to a story that did not include the protagonist’s active engagement in behavior control (*n* = 32; 15 girls). During the dictator game, half of the children were asked to share as many items as they wanted (*n* = 32; 15 girls), while the other half were asked to share as many items as they should with an anonymous partner (*n* = 32; 16 girls).

### Procedure

#### Introduction

First, Experimenter 1 gave the children instructions that described the procedure of the dictator game in which they would decide how to share stickers with an imaginary peer. Then, the experimenter told the children that someone else would come and play them a story about a child before the game. Finally, Experimenter 1 told the children that they could not listen to the story and left the room.

#### Priming

Experimenter 2 entered the room and greeted the participants. She then played the pre-recorded stories and presented accompanying pictures on a 22-in LED monitor (LG22MP58VQ). The audio clips for both conditions lasted 72 s.

The experimenter matched the sex of the story protagonist to that of the participants. The protagonist’s name was either Jae-ha, a Korean name that readily considered the boy’s name, or Jae-hui, a Korean name that readily assumed the girl’s name. The story of the male participants was as follows:

*This is Jae-ha. Jae-ha went to a room and found his favorite candies. The candies looked so delicious. Jae-ha wanted to eat the candies right away. However, his mother came and told him not to eat any because they would be having dinner soon. His mother said that he must not eat candies and he should wait for her to finish cooking dinner. She left the room and went to the kitchen.*

In the behavioral control prime condition, the story ended as follows:

*After his mother left, Jae-ha really wanted to eat the candies but thought he had to refrain from eating. The candies looked so sweet and delicious. However, Jae-ha did not eat any. Instead, Jae-ha sat in front of the candies and waited while his mother prepared dinner.*

In the neutral prime condition, the final part of the story was:

*After his mother left, Jae-ha left the room where the candies were kept and went to the living room. There were several toys there. Jae-ha decided to play with a LEGO set. So, Jae-ha built something with the LEGO blocks while his mother prepared dinner.*

When the stories ended, Experimenter 2 told the child that he would play a game next with another teacher. Then she left the room.

#### Emotion Rating

**Figure 1:**
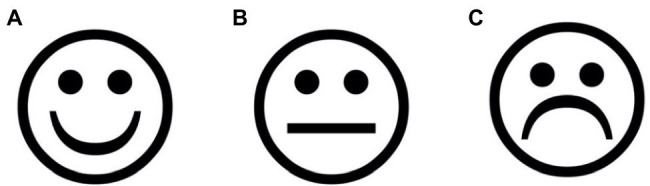
Pictures of emotional facial expressions used in Emotion Rating: **(A)** Good, **(B)** Neither good nor bad, and **(C)** Bad.

Experimenter 1 returned to the room and presented the child with printed line drawings of emotional facial expressions depicting “good,” “neither good nor bad,” and “bad” (Figure 1). Next, the experimenter asked the children to indicate which emotional expressions matched their own emotions at the moment. The experimenter coded their responses as 1 (*good*), 2 (*neither good nor bad*), or 3 (*bad*).

#### The Dictator Game

In the dictator game, we used stickers that sharing tasks widely utilized with 3–4-year-old children (Benenson et al., 2007; Gummerum et al., 2010; Smith et al., 2013). Experimenter 1 gave the children seven star-shaped stickers and two boxes. One of the boxes had the participant’s name and the other had no name. The experimenter instructed the children that the box which had their name belonged to them, and the box without a name belonged to an anonymous child. The experimenter then told them that they should divide the stickers between themselves and an unidentified child. The experimenter asked half of the children to share as many items as they wanted, and the other half to share as many items as they should. In addition, the researcher directed the children to count the number of stickers and indicate which box they belonged to and the recipient.

Experimenter 1 was unaware of the prime condition to which each participant was assigned because she was absent during the priming phase. This withheld information and reduced the likelihood of experimenter demand effects on the children’s responses during the dictator game task. Additionally, the experimenter told the children that they would not be observed while they shared. Instead, the experimenter’s gaze was directed toward the wall until the children signaled that they had finished.

#### Fairness Rating

After the dictator game task, Experimenter 1 told the children that another child had previously participated and had the chance to share seven stickers with an anonymous child. The other child shared as many stickers as they wanted. The experimenter then asked the children how many stickers they thought would have been fair for the other child to share.

## Results

**Figure 2:**
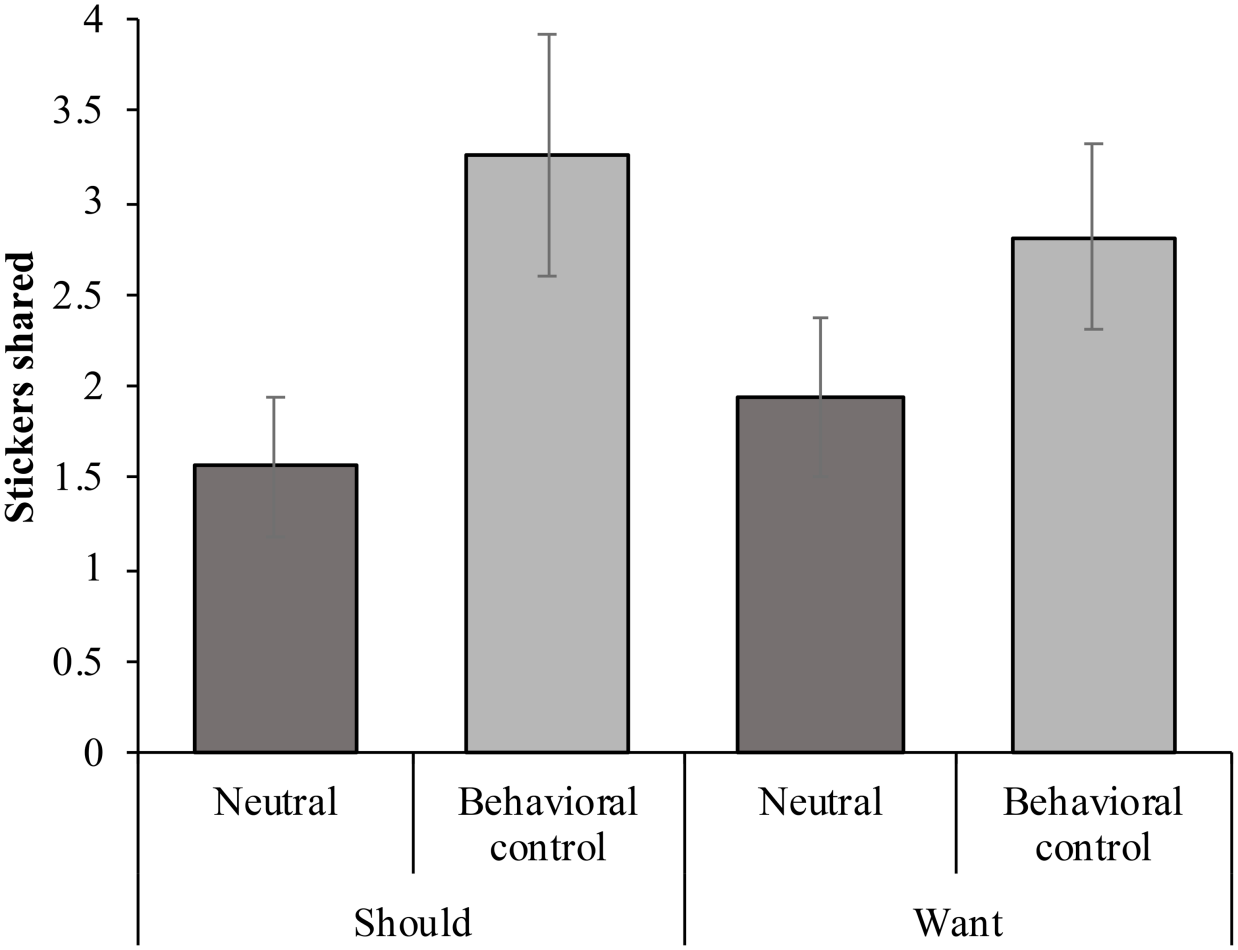
The mean number of stickers children shared during the dictator game per condition and sharing instruction. Error bars represent SEs.

The preliminary analyses showed no significant interaction between the priming and sex [*F*(1, 60) = 0.116, *p* = 0.735, partial $\eta^{2}$ = 0.002]. Therefore, we collapsed the data across sexes. We performed a two-way ANOVA to test the effect of the condition (behavioral control or neutral prime) and sharing instruction (want or should) on the number of stickers shared in the dictator game. A significant effect of condition emerged, suggesting that children in the behavioral control prime condition (*M* = 3.03, *SD* = 2.32) shared more stickers than children in the neutral prime condition (*M* = 1.75, *SD* = 1.61), *F*(1, 60) = 6.45, *p* = 0.014, partial $\eta^{2}$ = 0.097 (Figure 2). The effect of the condition remained significant after an analysis of covariance (ANCOVA) was used to control for emotion rating and fairness judgment [*F*(1, 50) = 6.547, *p* = 0.014, partial $\eta^{2}$ = 0.116].

There was no significant effect of sharing instruction [*F*(1, 60) = 0.004, *p* = 0.951, partial $\eta^{2}$ = 0.000], suggesting that the sharing of children told to share as they should [*M* = 2.41, *SD* = 2.28] did not significantly differ from that of children told to share as they wanted [*M* = 2.37, *SD* = 1.90]. The interaction between the sharing instruction and condition was not significant [*F*(1, 60) = 0.649, *p* = 0.424, partial $\eta^{2}$ = 0.011].

We tested for differences in emotion and fairness ratings between the behavioral control prime and neutral prime conditions using one-way ANOVA. There were no significant differences in emotional experience between the behavioral control prime (*M* = 1.19, *SD* = 0.471) and neutral prime conditions (*M* = 1.22, *SD* = 0.553) [*F*(1, 62) = 0.059, *p* = 0.808, partial $\eta^{2}$ = 0.001]. When analyzing differences in fairness judgment between conditions, we excluded nine children who did not answer the question and one whose response fell more than three *SD*s from the mean. There were also no significant differences in fairness judgments between the behavioral control prime (*M* = 4.70, *SD* = 2.02) and neutral prime conditions [*M* = 4.42, *SD* = 2.53; *F*(1, 52) = 0.193, *p* = 0.662, partial $\eta^{2}$ = 0.004].

## Discussion

The present study investigated the role of behavioral control in preschoolers’ sharing using a priming paradigm. Children primed with behavioral controls shared more stickers with anonymous recipients than those who did not undergo behavioral control priming. In line with previous research (Smith et al., 2013; Steinbeis and Over, 2017), there was no interaction between priming and sharing instruction. Thus, our 3- and 4-year-old participants primed by behavioral control shared more, not only when told to share at will, but also when told to share as they thought they should. That is, regardless of whether norms were highlighted, priming behavioral control had an effect on children’s sharing. The results suggest that preschoolers require behavior control when giving up a valuable resource, regardless of instruction.

This result is consistent with previous correlational findings suggesting a link between behavioral control and sharing in preschoolers (Aguilar-Pardo et al., 2013; Xie et al., 2019; Liang et al., 2020). Furthermore, the current study provides the earliest evidence on the causal role of behavioral control in enhancing preschoolers’ equal sharing by using an experimental manipulation in previous research on school-aged children (Steinbeis and Over, 2017). These results suggest that preschoolers’ mental representation of behavioral control can be activated by listening to a story about a person exerting behavioral control. We did not measure the exact level of children’s story comprehension. However, we assumed that children would have no trouble understanding the stories, because age-appropriate words and sentence structures were used and the stories were accompanied by pictures so as to aid comprehension. The results suggest that our participants must have understood the stories well enough to represent the protagonist’s behavioral control. Such an activated representation of behavioral control is abstract enough to guide young children to inhibit their selfish tendency and become prosocial in a later, unrelated task.

One key question is the process through which the activated representation of behavioral control promotes children’s sharing. One possibility is that priming behavioral control might change children’s emotional states, thereby influencing sharing. For instance, positive feelings are related to generous sharing among children (Moore et al., 1973; Underwood et al., 1977). If children felt better after hearing a behavioral control story than after hearing a neutral story, such differences in emotional experiences might be the reason for different sharing behaviors. The other possibility is that priming behavioral control might alter the judgment of fairness and guide sharing behavior. Children’s normative judgment of sharing is relevant to their sharing behavior (Paulus et al., 2018). However, there were no significant differences in emotional experience or fairness judgment in the current research between children who listened to the behavioral control story and those who listened to the neutral story. These findings allow us to exclude the possibility that priming behavioral control leads to generous sharing *via* changes in children’s emotional states or fairness judgments.

Third, priming behavioral control can increase the cognitive accessibility of abstract concepts regarding behavioral control. For example, thinking about someone with good self-control can make thoughts about various responses related to behavioral control more accessible. This self-control influences subsequent behavioral control task performance (vanDellen and Hoyle, 2010). Fourth, priming behavioral control can increase children’s motivation to adhere to social norms. For example, after listening to a story about a protagonist resisting temptation to disobey a rule, children might infer that the experimenters also expected them to exert behavioral control and behave in a socially acceptable way. Future research could examine the mechanisms (cognitive vs. motivational) that account for the effects of priming behavioral control on preschoolers’ sharing behaviors.

Notably, the neutral prime condition stories may not be completely neutral. The stories could have also primed the participants’ self-regulation but to a lesser degree than did the behavioral control prime. The act of leaving the room with the left candies could be viewed as an attempt to avoid further temptation. Future research could include a baseline condition in which the protagonist is not required to control their behavior; the data from such a baseline condition can then be compared with those of the neutral-prime condition. Such comparisons can inform us whether even very subtle, inexplicitly stated behavior control can prime children’s sharing actions.

Another open question is whether we could attribute the effect of priming behavioral control to the intention to exert behavioral control or to the successful outcome of behavioral control. For example, in the behavioral control story, the storyteller explicitly states the intention and outcome of behavioral control. The protagonist in the behavioral control story was intended to resist the temptation to eat treats and succeed. However, in a neutral story, neither the intention nor the outcome of behavioral control is explicitly stated. There was only a mere mention in the neutral story that the protagonist played with toys after leaving the treats. Therefore, it is difficult to tell whether priming the intention or outcome of behavioral control affects preschoolers’ sharing. Even priming an intention alone may affect children’s actions. Children aged 3 to 4 years who heard a story in which the protagonist intended to maximize rewards were more likely to choose larger and delayed rewards than children who heard a story in which the protagonist intended to receive immediate rewards (Kesek et al., 2011). Further research could explore whether priming an agent’s intention to exert behavioral control is sufficient or whether priming the successful outcome of behavioral control is required to enhance preschoolers’ sharing.

Why did sharing instructions not affect 3–4-year-old children’s sharing? Older children, 6–9 years of age, shared significantly more when asked to share as they should than when asked to share as they wanted (Steinbeis and Over, 2017). It may be possible that the present research was not sufficiently powered to detect small effects in children’s responses and that the present null effect of instructions on children’s sharing may be because of a lack of statistical power. However, this does not seem to be supported by the current results, which suggests a very small difference in means between the *want* and *should* conditions (the means were 2.41 vs. 2.37, respectively).

Another possible reason for this discrepancy between the previous and current findings is that 3- to 4-year-olds may have difficulty distinguishing norm-based sharing “should share” from desire-based sharing “want to share.” However, this is unlikely, given that children at this age can differentiate between what one should share and what one wants to share. For example, in Smith et al. (2013), when asked how they thought they should share with another child, 3- to 4-year-olds stated that they should share about half of the resources. Nevertheless, they anticipated that they would share less than half if they had the chance to share as they wanted. Moreover, 3–4-year-old Korean children are very likely to understand the meaning of the phrases because Korean children begin to produce “eo-ya” (a Korean morpheme whose meaning corresponds to “should” in English) and “siph-ta” (a Korean verb corresponding to “want to” in English) between 2 and 3 years of age (Lee et al., 2003; Pae and Kwak, 2011).

Another possibility is that although 3- to 4-year-olds can distinguish between the two instructions, they may be less likely to comply with norms in their actual behaviors. For example, McAuliffe et al. (2017) found that presenting children with sharing norms can affect sharing behavior, but the influence may differ with the age of the children. In that study, when the researchers provided the norm to give 80% of resources to another, 8–9-year-olds’ equal sharing behavior increased, whereas 4- to 5-year-olds still showed a selfish sharing bias. In other words, younger children’s pre-existing tendency for selfish sharing was less likely to shift with the explicit introduction of the generous norm. Likewise, in the current research, our instruction to share by following a norm (“as they should”) might not have been effective in increasing 3–4-year-olds’ altruistic sharing.

Questions can be raised regarding the lack of statistical significance in emotion and fairness ratings between the two conditions. As for emotion rating, note that we asked the children to rate their emotions immediately after priming to measure any subtle emotional state change, unlike Steinbeis and Over (2017), who asked the children about their emotional experiences after the sharing decision was made. However, we found no difference in the emotion ratings between the two conditions. This consistency between previous and current research supports the idea that priming selectively influences sharing actions, and not emotional experiences. However, there is also the possibility that emotion ratings consisting of three options might have been too insensitive to measure any differences in children’s emotional states. Future studies should examine this possibility.

The lack of condition differences in fairness judgment could be because the priming effect did not persist until the children answered their judgment of fairness. We asked the children about their fair judgment after participating in the dictator game. In contrast, previous research showed that school-aged children who were asked to share what they should share offered more resources to the other child than those asked to share what they would share in the dictator game (Blake et al., 2015). In the current research, we aimed to prevent explicit judgments about fairness from influencing sharing behaviors. Thus, we measured children’s fairness rating after making a sharing decision, as in Steinbeis and Over (2017). Such methodological features make it uncertain whether there is a short-term priming effect on fairness judgment, which could have affected children’s sharing behaviors. Future research could assess the influence of priming behavioral control on the judgment of fairness by asking about fairness judgments immediately after priming behavioral control.

This study contributes to ongoing debates on whether sharing arises from more intuitive, automatic, deliberate, and controlled processes. A dual-process model postulates that two broad processes interact to make decisions (Strack and Deutsch, 2004). The first type, the automatic process, is fast, unconscious, and effortless. The second type, the controlled process, is slow, partly conscious, and effortful (Schneider and Shiffrin, 1977; Alós-Ferrer and Strack, 2014). Therefore, we can use this model to address the key question of whether sharing behavior relies more on automatic or controlled processes. Studies of adults have yielded mixed results. Some studies have shown that sharing decisions occur automatically (Rand et al., 2012; Cappelen et al., 2016), while others have found that sharing decisions require effortful processes (Achtziger et al., 2015). Consistent with previous findings (Liang et al., 2020), the current research suggests that controlled processes, such as behavioral control, may play an important role in altruistic sharing during early childhood and school years (Steinbeis and Over, 2017). Neuroscientific research has also provided evidence that preschoolers’ sharing may rely more on controlled processes. When 3–5-year-old children watched characters engaging in prosocial or antisocial behaviors, individual differences in the later controlled patterns of neural responses, but no differences in the early automatic patterns, predicted children’s sharing (Cowell and Decety, 2015).

In conclusion, the present research adds to the growing body of literature that explains the gap between fairness understanding and sharing behavior in early childhood. Young children may fail to share equally with others because of their difficulty in behavioral control. Furthermore, our findings have important implications for interventions that promote sharing behaviors during early childhood. Sharing in young children can be enhanced by priming behavioral control. Future studies could examine the effectiveness of interventions to promote sharing behavior in young children by priming behavioral control *via* media, such as storybooks or videos.

## Data Availability Statement

The raw data supporting the conclusions of this article will be made available by the authors, without undue reservation.

## Ethics Statement

The studies involving human participants were reviewed and approved by Yonsei University IRB. The patients/participants provided their written informed consent to participate in this study.

## Author Contributions

H-JS developed the concept and design of the study and provided critical revisions. CL performed data collection and analysis and wrote the first draft of the manuscript. All authors contributed to the article and approved the submitted version.

## Funding

The Ministry of Education of the Republic of Korea and the National Research Foundation of Korea (NRF-2018S1A 3A2075114) supported this work.

## Conflict of Interest

CL was employed by Assesta Co., Ltd.

The remaining author declares that the research was conducted in the absence of any commercial or financial relationships that could be construed as a potential conflict of interest.

## Publisher’s Note

All claims expressed in this article are solely those of the authors and do not necessarily represent those of their affiliated organizations, or those of the publisher, the editors and the reviewers. Any product that may be evaluated in this article, or claim that may be made by its manufacturer, is not guaranteed or endorsed by the publisher.
